# ASCO Congress 2018: melanoma treatment

**DOI:** 10.1007/s12254-018-0455-4

**Published:** 2018-11-20

**Authors:** Erika Richtig

**Affiliations:** Department of Dermatology, Medical University of Graz, Auenbruggerplatz 8, 8036 Graz, Austria

**Keywords:** Melanoma, Merkel cell carcinoma, Non-melanoma skin cancer, New targets, Brain metastases

## Abstract

The 2018 ASCO Annual Meeting provided a closer look on the details of studies already presented. In melanoma, the interest was on neoadjuvant treatment options with high pathological response rates as well as updates on large phase III studies in stage IV disease. Further new targets were discussed focusing on additional drugs to a PD-1 backbone treatment.

Another focus was on Merkel cell carcinoma and basal cell carcinomas, giving new data on PD-1 antibody treatments as well as on vismodegib as neoadjuvant therapy.

## Melanoma

### Stage III and adjuvant treatment

#### Lymph node dissection

Lymph node dissection (LND) after positive sentinel lymph node biopsy (SLNB) has been a long-lasting practice in surgery for melanoma patients. At the ASCO Congress, the final data of the DeCOG-SLP Study were presented by Ulrike Leiter. In this study, 483 patients with positive SLNB were randomized, 241 in the observation arm and 242 in the LND arm. Median follow-up was 72 months with a hazard ratio of distant metastasis-free survival of 1.08 and hazard ratio for overall survival (OS) of 0.99 (cut-off 1 February 2018). Prognostic factors for distant metastasis-free survival and OS as well as recurrence-free survival were tumor load in the SLNB and tumor thickness [1].

LND after positive SLNB is therefore no longer state of the art. Discussions remained around melanomas of the head and neck not included or only minimally included in the DeCOG-SLP Study presented or the previously published MSLT-II trial.

#### Adjuvant treatment

In 2017 Georgina Long et al. published in *The New England Journal of Medicine* the 3‑year relapse-free and OS rates for adjuvant treatment with dabrafenib and trametinib with a hazard ratio of 0.47 and 0.57, respectively, but with 41% of grade 3 or 4 toxicities, mostly fever. The best results were obtained in patients with normal lactate dehydrogenase (LDH) and low tumor volume. All side effects were fully reversible after terminating the treatment.

During the ASCO, adjuvant immunotherapy was discussed taking several studies into consideration: EORTC 18081 (ipilimumab vs. placebo), CheckMate 238 (ipilimumab 10 mg per kilogram vs. nivolumab), EORTC 1325 (pembrolizumab vs. placebo). All immune checkpoint inhibitors showed better relapse-free and OS vs. placebo, and pembrolizumab and nivolumab showed better outcome compared with ipilimumab [2, 3]. The consensus was that patients with BRAF wild-type melanoma should receive immunotherapy in the adjuvant setting, BRAF-mutant patients should be offered both options: PD1-blockade as well as dabrafenib and trametinib. Toxicities in the adjuvant PD-1 studies were lower than with dabrafenib and trametinib.

The discussion focused on stage IIIA disease: It seems that consideration of the tumor load in SLNB might play a role with a cut-off of 1.0 mm. Patients with tumor load below 1.0 mm in the SLN had a better outcome than patients with tumor load over 1.0 mm. This could be helpful when thinking about an adjuvant treatment decision [4].

#### Neoadjuvant treatment

Neoadjuvant treatment with ipilimumab + nivolumab vs. nivolumab was discussed showing impressive response rates, but 73% grade 3–4 toxicities were found in the combination arm. The response rate was 73%, with 45% pathological complete remissions (CR). In the nivolumab arm there were 25% response rates, in 25% pathological CR [5].

A randomized phase II study with talimogene laherparepvec + surgery vs. surgery alone in stage IIIB–IV M1a melanomas was presented. Talimogene laherparepvec, genetically modified oncolytic herpes simplex virus, was given once with up to 4 ml 10^6^ PFU/ml and followed after 3 weeks by up to 4 ml 10^8^ PFU/ml every 2 weeks. Surgical intervention took place after 13 weeks. In the observation arm, surgery was performed immediately. A total of 75 patients were randomize to each arm. In about 15% of patients, CR and partial remission (PR) was observed, while in 30% there was stable disease (SD) [6].

### Melanoma stage IV

#### Studies update

Columbus Study (phase III study with encorafenib + binimetinib vs. vemurafenib or encorafenib in BRAF-mutant melanomas): Data were presented after 18 months of follow-up. Median OS in the combination arm was 33.6 months, for vemurafenib, 16.9 months. The 1‑year OS for the combination was 76%, for vemurafenib, 63%. After 2 years, OS for the combination was 58%, for vemurafenib it was 43%; after 3 years, there was a 47% OS rate for the combination arm and 32% for the vemurafenib arm. Subgroup analyses with elevated LDH and more than three organs affected revealed lower differences. OS in the combination arm versus encorafenib alone was 33.6 months versus 23.5 months. Median progression-free survival (PFS) for the combination arm was 14.9 months, for encorafenib alone it was 9.6 months, and for vemurafenib alone it was 7.3 months. Overall response rate (ORR) was 64%. Median duration of response was 18.6 months for the combination arm. Adverse events were reported in 98–100% of cases in all three arms; grade 3 and 4 adverse events were distributed equally in all three study arms at 64%, 67%, to 66%, respectively [7].

Data from the KEYNOTE-006 study on 4‑year survival after ending 2 years of pembrolizumab treatment were reported. In the study, pembrolizumab was given versus ipilimumab in treatment-naïve metastatic melanoma patients, only BRAF-mutant patients were allowed to have one previous treatment. Median-follow up was 45.9 months. The study was randomized 1/1/1 to pembrolizumab 10 mg every 2 weeks vs. pembrolizumab 10 mg every 3 weeks and ipilimumab 3 mg/kg every 3 weeks for up to four doses. Pembrolizumab was given for up to 2 years. After 4 years, OS in the pembrolizumab arm was 41.7% and in the ipilimumab arm it was 34.1%. In treatment-naive patients, OS in the pembrolizumab arm was 44.3% and in the ipilimumab arm it was 36.4%. Median PFS for pembrolizumab was 8.3 months, for ipilimumab it was 3.3 months; in treatment-naive patients, for pembrolizumab it was 11.2 months and for ipilimumab, 3.7 months. ORR for pembrolizumab was 42% and for ipilimumab 17%; in treatment-naive patients it was 47% for pembrolizumab and 17% for ipilimumab.

Of a total of 556 patients having received pembrolizumab, 103 were treated for 2 years. Out of these patients, 28 were in CR, with 26 patients remaining in CR for another 2 years, while two patients had progressive disease (PD); three patients received a second cycle of pembrolizumab. PR was observed in 65 patients, of whom 56 remained in PR; nine patients (13.8%) experienced PD and four of them had a second cycle. Out of ten patients with SD, seven remained in this stage after another 2 years. Three patients (30%) had PD and one patient had a second cycle of pembrolizumab. Out of eight patients who had a second cycle of pembrolizumab, only one had further progression [8].

### Brain metastases and radiotherapy

Another focus was the management of brain metastases and surgery as well as stereotactic radiosurgery versus systemic treatment and combinations of these options were discussed. There was a high level of consent that treatment of brain metastases has to be individualized differentiating between symptomatic and asymptomatic patients, number of lesions in the brain, their size and localization, BRAF mutation status, status of extracranial metastases, and the dynamic of progression.

A combination of immune checkpoint treatment with stereotactic radiosurgery showed better OS in patients compared with ipilimumab alone (hazard ratio: 0.43). Another review that was presented showed in 75 patients that concurrant immune checkpoint treatment and stereotactic radiosurgery had better response rates than patients receiving these two treatment options in sequence [9].

### IDO inhibitors

The KEYNOTE-252 study comparing epacadostat + pembrolizumab vs. pembrolizumab alone in metastatic melanoma reported negative results concerning PFS and OS despite encouraging data in the phase I and II studies and was thus stopped.

At ASCO, data of the ECHO-204 study were presented. This was a phase II study evaluating the combination of epacadostat + nivolumab in different tumor entities. ORR was 62%, with CR in 18% of patients. Treatment response was even higher at 75% in PDL-1-positive patients and 75% in IDO-positive patients. In total, 60% of the treated patients were alive after 1 year. Adverse events were similar to those for PD-1 monotherapy.

The last study presented with an IDO inhibitor was the phase II study NLD-2103 with indoximod in combination with pembrolizumab in 85 patients. ORR was 53%, there was CR in 18% of patients with approximately the same toxicities as those reported in PD-1 monotherapy. Patients with a positive PD-L1 status were more likely to respond to treatment (77%).

Unfortunately, all studies with IDO inhibitors were stopped and the future of IDO inhibitors remains unclear especially as biomarkers are missing [10].

### TLR9 agonists

The ILLUMINATE-204 study was also presented. In this study, tilsotolimod, an oligonucleotide stimulating the toll-like receptor 9 (TLR9) was injected intratumorally and combined with ipilimumab. All patients had a prior treatment with CTLA-4 inhibitor, PD-1 inhibitor, or a combination of CTLA-4 and PD-1. The response rate was 38% in 21 patients, the disease control rate, 71%. A phase III study is planned. In addition, a poster including relevant data was also presented.

Another study focusing on TLR9-agonist SD-101, also given intralesionally, was presented. In this study the SD-101 was combined with pembrolizumab at a fixed dose of 200 mg. Phase IB was started with a dose escalation of 1–8 mg. In phase II, doses of 2 mg in combination with pembrolizumab 200 mg as well as 8 mg in combination with pembrolizumab 200 mg were applied. The dose of 2 mg could be administered in up to four lesions, 8 mg only in one lesion. Anti-PD-1-antibody-naive patients as well as patients whose disease progressed under anti-PD-1 treatment were eligible. ORR in the 2‑mg cohort was 70% and in the 8‑mg cohort it was 38%. The 6‑month PFS was 76% for the 2‑mg group and 41% for the 8‑mg group [10].

### New targeted therapies

Focus was directed on the MITF gene (melanocyte inducing transcription factor). MITF is a protein-coding gene playing a substantial role in the development of melanocytes and in the regulation of the expression of Tyrosinase and tyrosinase-related protein 1. MITF-low cell status was shown to be associated with resistance in MAP-kinase pathway inhibition. MITF-low tumors are also found in uninflamed tumors as well as in non-responders to immune checkpoint therapy. One possible treatment target could be the change of a low MITF status in cells to a high status. Inhibitors of the oxidative phosphorylation or JAK inhibitors might play a role [11].

A phase I/II study called Pivot focusing on the combination of NKTR-214 (CD-122 biased agonist) with nivolumab in patients with metastatic solid tumors was presented. NKTR-214 modifies the interleukin 2 pathway with an augmented proliferation of tumor-infiltrating lymphocytes and higher PD-1 expression on the surface of CD8-positive T‑cells. The Pivot-02 Study was a dose escalation study of immune checkpoint inhibitor therapy-naive patients. The ORR was 64%, and the disease control rate (DCR) was 91%. Phase II studies were developed even further for five tumor entities (melanoma, renal cell carcinoma, non-small cell lung cancer, urothelial cancer, and triple-negative breast cancer). In melanoma stage IV disease in (immuno-oncology) I‑O treatment native patients, ORR was 85% and best overall response rate (BORR) in phase II was 50%. The grade 3/4 side effects reported were pneumonitis, skin reactions, hepatitis, colitis, elevated lipase, and diabetes and hyperglycemia. Nivolumab was given at a fixed dose of 240 mg every 2 weeks [12].

### Treatment sequences

Christian Blank reported about a retrospective single-center analysis by Reijers, Rozeman, and Blank on whether or not survival would be better if a targeted therapy was switched very early to an immune checkpoint inhibitor treatment. In this study, OS was significantly better (*p* = 0.007) when switching the treatment without progression.

Another study, focusing on treatment switches in BRAF-mutant melanoma patients is the currently recruiting SECOMBIT study: arm A on encorafenib and binimetinib until progression and then switching to ipilimumab and nivolumab; arm B on ipilimumab and nivolumab until progression and then switching to encorafenib and binimetinib; and arm C on encorafenib and binimetinib for 8 weeks and then switching to ipilimumab and nivolumab until progression and then encorafenib and binimetinib upon progression.

Another study in this field is the COWBOY study in BRAF-mutant melanoma patients. Randomization is 1:1 to vemurafenib and cobimetinib followed by ipilimumab and nivolumab with a nivolumab maintenance therapy. The second arm is ipilimumab and nivolumab for four cycles followed by nivolumab alone.

### Further new targets

The following targets were presented:

ICOS is a co-stimulatory signal on T‑cells expressed if a T-cell has contact with an antigen-presenting cell. Phase I and II studies with JTX-2011 as monotherapy as well as in combination with nivolumab are under investigation.

CD27 is another co-stimulatory signal on the surface of T‑cells in contact with antigen-presenting cells. Under the name of varlilumab it has been tested in early phases in combination with nivolumab especially in ovarian cancer patients. Further tests will be made in MSI-H/TMB high colorectal cancers.

CD47 is a molecule on the surface of tumor cells inhibiting phagocytosis through macrophages. CD47 also influences antigen presentation. This substance is tested in early stages of colorectal and ovarian cancer.

### Novel treatment combinations

Immunotherapy and immunotherapy: possible combinations are T‑VEC + ipilimumab or TVEC + pembrolizumab. Further substances are PV-10 (rose bengal), CVA-21 (Coxsackie virus A21), PIL-12 (plasmid IL-12 and electroporation), LTX-315 (peptide, derived from lactoferrin), TLRs, STING agonists, and various others.

Combinations under investigation are anti-PD-1/PDL-1 components with anti-GITR, anti-LAG-3 (e. g., relatlimab), and HDAC inhibitors (entinostat).

Concerning LAG-3, a double-blind phase II/III study, CA224-047, combining nivolumab and relatlimab vs. nivolumab as first-line therapy is recruiting. Further anti-LAG-3 substances under investigation are: LAG 525 (Novartis), NK-4280 (MST) to name just two of them. HDAC inhibitors entinostat and pembrolizumab are tested in a study in metastatic melanoma patients after progression during or after a PD1/L1 antibody therapy. A triple combination with the aforementioned substances could be the combination of HDAC inhibitor + anti-PD-1 + anti-LAG-3. At ASCO 2017, a phase I/IIa study was presented with anti-GITR in combination with nivolumab led to increased proliferation and activation of CD8-positive cells in patients with solid tumors.

Immunotherapy and targeted therapy: several studies are under investigation, including dabrafenib + trametinib in combination with durvalumab, dabrafenib and trametinib in combination with pembrolizumab, vemurafenib + cobimetinib with atezolizumab, as well as dabrafenib and trametinib in combination with spartalizumab.

Furthermore, another phase III study testing the combination of atezolizumab with cobimetinib for BRAF wild-type patients is under investigation.

Substances focusing on metabolism were also presented:

- Amino acid metabolism: e. g., lenalidomide.
- Lipid metabolism: e. g., galloflavin.
- Oxfoss: e. g., metformin and potentially sorafenib.
- Adenosine metabolism: anti-CD73 could be a possible target. A phase I study with anti-CD73 in combination with nivolumab is underway for solid tumors [13, 14].

### Poster

An update on the 5‑year survival rate for the KEYNOTE-001 study was presented, with 5‑year PFS of 21% and OS of 34% in all patients and 29 and 41%, respectively, in treatment-naïve patients. Response was durable in 73% of the total population and in 82% of the treatment-naïve patient group. Median DOR was not reached in both groups [15].

## Non-melanoma skin cancer

### Merkel cell carcinoma

An update of the JAVELIN Merkel 200 study Part A was presented, giving the 2‑year efficacy and safety data. In this study, avelumab versus chemotherapy was evaluated in patients with metastatic disease with progression after chemotherapy. The median follow-up was 29.2 months, median duration of treatment was 3.9 months. ORR was 33% and DCR 43.2%. PFS after 1 year was 29%, after 2 years, 26%. The 1‑year OS was 50%, and the 2‑year OS was 36%. Side effects were observed in 67% of patients; grade 3/4 side effects were seldom. Neither Merkel cell polyoma virus nor PD-L1 expression was a predictive marker of response [16].

CheckMate 358 (nivolumab, neoadjuvant in patients with resectable Merkel cell carcinoma) was presented. Prior to surgical removal, treatment with nivolumab 240 mg day 1 and day 15 was administered followed by surgery on day 29. After surgery, only follow-up according to the national guidelines was performed. In 65% of 29 patients (17 patients) pathological CR or major pathological remission was achieved [17].

Finally, data on pembrolizumab as first-line therapy in metastatic Merkel cell carcinoma were given. Pembrolizumab 2 mg/kg every 3 weeks was given up to 2 years. Out of a total of 50 patients, 50% showed a response independent of the virus stage. Response rates were long lasting at presentation (median DOR not reached). Median PFS was 16.8 months in comparison with previously published data for chemotherapy with PFS between 3.1 and 4.6 months [18].

In the ensuing discussion, it was mentioned that patients with chemotherapy prior to immune checkpoint therapy had worse outcome. Therefore, anti-PD-1 or anti-PD-L1 should be given as first-line therapy.

Adjuvant treatment after resection of Merkel cell carcinoma with ipilimumab showed no benefit (poster by Becker et al. ADMEC study: 19 patients in the treatment arm and 17 patients in the observation arm; no difference in disease progression between the two arms, and the study was closed at an early stage).

### Basal cell carcinoma

Neoadjuvant vismodegib (VismoNeo Study: vismodegib 150 mg per day) showed response in 80% of the patients; of the remaining patients, ten had SD and one patient had PD. In 49% of the patients, a complete pathological remission was obtained [19].

## Conclusion

PD-1 antibodies remain the backbone of melanoma treatment in immunotherapy of metastatic melanoma. Data on BRAF and MEK inhibitor therapy showed excellent response rates and good OS data for V600-mutant patients.

Despite new targets presented herein, the question about sequencing immunotherapy and targeted therapy in BRAF V600-mutant melanoma patients remained unsolved.

In metastatic Merkel cell carcinoma, immunotherapy is state of the art in first-line treatment.
